# Barron’s rubber band ligation vs. *Kshar Sutra* ligation

**DOI:** 10.4103/0974-7788.72496

**Published:** 2010

**Authors:** Viroj Wiwanitkit

**Affiliations:** *Wiwanitkit House, Bangkhae, Bangkok Thailand 10160, Bangkok E-mail: wviroj@yahoo.com*

Sir,

I read the recent publication on ligation by Singh *et al*.[1] with great interest. The authors concluded that “*Kshar Sutra* ligation is a useful form of treatment for Grades II and III internal hemorrhoids.”[1] I agree that this report might imply some clinical usefulness of *Kshar Sutra* ligation. The thread that is used in *Kshar Sutra* therapy is confirmed for active compositions in ethnopharmacology.[2] However, there are some points to be further discussed. First, there are a few subjects studied, which might limit the usefulness of the results. The clarification on statistical acceptability is needed. Second, it is interesting to know the comparative cost effectiveness between both ligation techniques. Third, the comparison on the operative time and attempt as well as acute and long-term complications rate should also be done. To fulfill the present study, these information are required.
